# Do we behave differently on Twitter and Facebook: Multi-view social network user personality profiling for content recommendation

**DOI:** 10.3389/fdata.2022.931206

**Published:** 2022-08-03

**Authors:** Qi Yang, Aleksandr Farseev, Sergey Nikolenko, Andrey Filchenkov

**Affiliations:** ^1^Machine Learning Lab, ITMO University, St. Petersburg, Russia; ^2^Somin Research, SoMin.AI, Singapore, Singapore; ^3^Steklov Institute of Mathematics at Saint Petersburg, St. Petersburg, Russia

**Keywords:** user profiling, multimedia retrieval, machine learning, recommender systems, user personality profiling, multimodal retrieval

## Abstract

Human personality traits are key drivers behind our decision making, influencing our lives on a daily basis. Inference of personality traits, such as the Myers-Briggs personality type, as well as an understanding of dependencies between personality traits and user behavior on various social media platforms, is of crucial importance to modern research and industry applications such as recommender systems. The emergence of diverse and cross-purpose social media avenues makes it possible to perform user personality profiling automatically and efficiently based on data represented across multiple data modalities. However, research efforts on personality profiling from multi-source multi-modal social media data are relatively sparse; the impact of different social network data on profiling performance and of personality traits on applications such as recommender systems is yet to be evaluated. Furthermore, large-scale datasets are also lacking in the research community. To fill these gaps, in this work we develop a novel multi-view fusion framework PERS that infers Myers-Briggs personality type indicators. We evaluate the results not just across data modalities but also across different social networks, and also evaluate the impact of inferred personality traits on recommender systems. Our experimental results demonstrate that PERS is able to learn from multi-view data for personality profiling by efficiently leveraging highly varied data from diverse social multimedia sources. Furthermore, we demonstrate that inferred personality traits can be beneficial to other industry applications. Among other results, we show that people tend to reveal multiple facets of their personality in different social media avenues. We also release a social multimedia dataset in order to facilitate further research on this direction.

## 1. Introduction

Over the past decade, an increasing number of social media platforms have been rapidly emerging; these platforms are already playing a vital role in facilitating human interactions worldwide. Since 2012, the average daily social media screen time has increased from 60 to 144 min (MIN, 2022). Furthermore, it has spiked even higher since the beginning of the COVID-19 disease outbreak (Farseev et al., 2020a), when many people around the world have been locked at home with the only remaining option of engaging their friends through social media and videoconferencing.

To maintain high user engagement rates, it is essential for social network conglomerates to position and recommend relevant content according to user interests and online behaviors. For example, extroverted people are more likely to use social media in general as they tend to reveal themselves as enthusiastic, interactive, and therefore forming more social circles around themselves (Gil de Zúñiga et al., 2017). On the other hands, introverts have been found to be spending significantly more time evaluating the value of each online service they use before a deeper user-service interaction may occur (Lu and Hsiao, 2010).

With such large and diverse data available nowadays on social media, it is getting virtually impossible to manually distinguish social media users when attempting to provide them with more personalized online experiences (Farseev et al., 2018). This leads to the need for an automated approach to human behavior pattern understanding on social media (Farseev et al., 2020b). However, nowadays personality profiling still heavily relies on manual procedures such as questionnaires and quizzes (Murray, 1990), which require an especially high level of cooperation from the user. Therefore, the cost of personality profiling remains unacceptably high for wide application, limiting its use in real-time online services such as social networking websites (Farseev et al., 2016).

Automated recognition of personality traits is known to be a hard problem (Buraya et al., 2018; TWI, 2022), mainly due to the multi-faceted nature of social media data. For example, *Twitter* is often used for casual daily interactions, while *Facebook* is currently perceived more as a private communication channel. As a result, *Facebook* audience demographics varies drastically, spanning both young and senior age groups, while, e.g., *TikTok* audiences mostly consist of young individuals, between 18 and 34 years old. Yet other social networks might not just have a non-uniform age distribution in their audience but also other characteristic features, e.g., *Pinterest* tends to be largely populated by female users (PIN, 2022).

These demographic considerations can be supplemented and significantly improved by exploring the drastic difference in behavioral traits that people exhibit across various social media avenues. For example, *Twitter*, being one of the most open social media outlets, is known to concentrate on the users' self-expression rather than their identity and capture more about the users' public personality intended for the broader public (TWI, 2022). On the other hand, specialized personality-focused forums such as *PersonalityCafe*^1^ may concentrate their communication on the members' behavioral habits, allowing to gain a deeper insight into a user's behavior from the content they post. In such a multi-faceted multi-source cross-demographic environment, the task of automated personality profiling from social media data appears to be both highly relevant and very challenging. Since this topic has not been widely explored by the research community yet, it requires a more in-depth analysis; in this work, we make the first steps in such an analysis.

Personality trait recognition has many applications, e.g., related to user retention, increasing user engagement, and the like. One of the most important classes of such applications deals with recommender systems that are able to leverage known user personality traits to further improve recommendations (Dhelim et al., 2021). Recommender systems are key parts of any social network, used both in order to increase user engagement *via* recommending useful and interesting content and in order to increase the profit of the network itself through more relevant advertising. In this work, we also consider recommender systems as a key application validating the usefulness of our approach.

Despite the advantages of leveraging multiple data modalities and sources, there are several important difficulties that we identify for the personality recognition problem:

1. **Data gathering**: data from modern social media platforms is often distributed across various online resources and shielded behind privacy settings; it is therefore important to implement large-scale cross-source data collection techniques;
2. **Data representation**: since real-world social media data comes with different data modalities (such as text, image, video, location etc.), to incorporate such heterogeneous multi-modal data requires us to implement mutually compatible state of the art approaches to data representation (feature learning);
3. **Data modeling**: efficient data integration into a single machine learning model is a challenging task, as the data sources and data modalities often represent various aspects of human life and therefore are often very different in nature; moreover, the high dimensionality of multi-modal feature spaces often leads to the so-called “curse of dimensionality” problem when being processed directly, and therefore dimensionality balancing needs to be accomplished.

Inspired by the research gap and challenges outlined above, in this work we raise four main research questions.

RQ1 **Is it possible to reliably and accurately infer user personality traits at scale in an automatic fashion?** This is the basic question of our study and a key question to establish a benchmark for multi-view personality profiling.
RQ2 **Is it possible to improve personality profiling performance by leveraging multi-view social multimedia data?** This question is important to assess the real world applicability of our personality profiling approach to modern social media scenarios, where information about users may come from many different sources and modalities, and different users may have crucial data in different modalities.
RQ3 **Is it possible to improve the performance of recommender systems by introducing additional features related to automatically inferred personality traits?** This is a key question to understand the real life applicability of our research, as recommender systems are a key part of social networks, and any positive impact of the user personality signal on their performance would have significant real world ramifications.
RQ4 **What is the impact of social media data origin on the performance of user personality profiling?** This is needed to establish a clear path of future research on multi-source and multimodal learning.

To answer our proposed research questions, in this study we introduce a novel multi-view personality profiling meta ensemble framework, called PERS, which is able to efficiently profile social media user personality by leveraging multimodal multimedia data coming from multi-faceted social networks. Then we evaluate the performance of recommender systems leveraging the personality signal, showing that user personality is beneficial for state of the art personalized content recommender system. Furthermore, we introduce efficient data gathering and representation techniques, allowing for seamless processing of data from *Facebook, Twitter*, and *PersonalityCafe* social media forums. Finally, we release the PERS dataset^2^ to the research community, allowing for future extensive cross-disciplinary research; we view this dataset, described in detail in Section 3.2, as an important contribution of the present work.

The major contributions of this work are four-fold. First, we propose a novel machine learning framework for multi-view user profiling and demonstrate that efficient personality profiling is possible, achieving industry-level performance for several personality attributes. Second, we demonstrate that different social networks are different in nature, which impacts the personality profiling performance and therefore needs to be considered during the data modeling process. Third, we demonstrate that the user personality signal is beneficial to the performance of state of the art recommender systems, with immediate consequences for real world applications of our model. Fourth, we present and release to the public a new multi-source cross-social personality profiling dataset designed to allow further research on personality traits in social network analysis and recommender systems.

## 2. Related work

The past three decades have seen several studies attempting to model human personality traits from a statistical perspective. First, the *Big Five* model was proposed by Digman (1990); the author revealed a close relationship between human personality and their written language, inferred from statistical analysis of the English lexicon. Inspired by the idea, Pennebaker and King (1999) later laid the foundation of statistical personality profiling by introducing the LIWC word categorization scheme that has numerically bridged personality traits and written language utilization patterns.

Several studies have been devoted to automatic personality profiling, where cross-disciplinary research groups were utilizing machine learning techniques for automatic human personality inference based on test-generated data (Argamon et al., 2005; Mairesse et al., 2007). We note, however, that these studies are all based on relatively small datasets and therefore have limited possibilities to extend to large-scale datasets that would appear in a real-world scenario. Moving forward, the problem with insufficient data was partially mitigated by the introduction of the “MyPersonality” project (Kosinski et al., 2015), the first large-scale personality-labeled dataset that includes user-generated data from *Facebook*; this dataset immediately attracted multimedia community attention, facilitating the first larger-scale studies in social media personality profiling research (Gjurković and Šnajder, 2018; Tadesse et al., 2018; Kumar and Gavrilova, 2019).

These studies have made a big leap in the field, but one could also notice that most of them still lack a very important factor limiting their real-world applicability: they are largely focused on a single data source (e.g., *Facebook*) or a single data modality (e.g., text). On the other hand, modern social network data is multi-source, multi-view, and multimodal. In particular, the Linguistic Inquiry and Word Count (LIWC) works are mostly focused on text-only data processing to predict personality by using personality-labeled word categories (Holtgraves, 2011; Sumner et al., 2012), while Arnoux et al. (2017) and Tandera et al. (2017) instead utilized pretrained GloVe embeddings of text data (Pennington et al., 2014) and were the first to report the results of machine learning-driven unimodal personality inference.

Finally, there were several studies that approached user profiling from a multimodal data perspective. For example, Farseev et al. (2015) proposed a multimodal ensemble model for the demographic profiling problem from multimodal data. Later, Farseev and Chua (2017a) extended the framework to leverage sensor data and multi-source multi-task learning for wellness profiling. Buraya et al. (2017) proposed to solve the problem of relationship status inference by applying ‘out of the box” machine learning on early-fused data from *Twitter, Instagram, Facebook*, and *Foursquare*, achieving a significant 17% increase in performance compared to unimodal learning. Going further, Tsai et al. (2019) proposed a factorization method to model the intra-modal and inter-modal relationships within multimodal data inputs, which proved to be important for the incorporation of multimodal data into user profiling, while Buraya et al. (2018) instead leveraged the temporal component of the multimodal data, being the first to apply deep learning methods for multi-view personality profiling. While multimodal data has already been tackled in these works, all of them still lack multi-source cross-social network data processing (Farseev, 2017), which limits their applicability in the majority of real-world scenarios.

As we have seen, there is significant evidence that incorporation of multi-modal data for automatic user profiling is useful to achieve better prediction performance. However, when it comes to evaluating the role of social network choice for user profile learning, existing research results remain to be relatively sparse. At the same time, it is reasonable to assume that often serving different needs of an individual, various social media sources might provide data that is very diverse in nature, and therefore a more comprehensive study on the roles of different data sources for personality user profiling is necessary.

Another direction of study that we improve with extracted personality traits are content recommendation systems. In classical collaborative filtering, matrix factorization (Bell et al., 2007; Mnih and Salakhutdinov, 2007; Koren and Bell, 2011) has become the standard baseline, with a huge number of variations and applications; it is still competitive but there are alternative approaches. Autoencoder-based models learn a functions that maps user feedback to user embeddings instead of learning the embedding matrix explicitly. Early approaches utilized shallow autoencoders (Sedhain et al., 2015; Wu et al., 2016), while variational autoencoders allowed to train more complex and deep models (Liang et al., 2018; Kim and Suh, 2019; Lobel et al., 2019; Mirvakhabova et al., 2020; Shenbin et al., 2020). Another recent class of models in collaborative filtering is based on graph convolutional network, including NFCF (Wang et al., 2019) and LightGCN (He et al., 2020) that are computationally heavy but demonstrate impressive performance, with more recent approaches such as GF-CF (Shen et al., 2021) and UltraGCN (Mao et al., 2021) improved both performance and computational efficiency. In this work, we specifically need models that can take into account new features such as personality traits; we selected the Personalized Content Discovery (PCD) model (Gelli et al., 2018) because it was tailored to a similar problem of content discovery for brands but also note several other works that extend recommender systems with extra features and extra data modalities (Zhang et al., 2016; Tanjim et al., 2020; Gao et al., 2021; He et al., 2021; Cai et al., 2022).

## 3. Data and feature extraction

### 3.1. MBTI personality categorization

To represent human personality, in this work we use the Myers-Briggs Type Indicator (MBTI) (Myers, 1998), widely adopted by the research community (Buraya et al., 2017, 2018). MBTI splits human personality into 16 types, each formed by the following four binary dimensions:

- **E**xtroversion vs. **I**ntroversion (EI): this dimension determines how an individual focuses her energies and interest, whether she is influenced externally by the opinions and interpretations of others (extroverts) or motivated by her inner thoughts (introverts).
- **S**ensing vs. i**N**tuition (SN): this aspect demonstrates how people interpret knowledge. Sensing personalities make decisions based on their five senses and solid observation, whereas intuitive individuals favor imagination to constancy.
- **T**hinking vs. **F**eeling (TF): a person with the thinking aspect prioritizes logical behavior in their decisions, while feeling individuals are empathic and give priority to emotions over logic.
- **J**udging vs. **P**erceiving (JP): this dichotomy describes an individual approach toward work, decision-making, and planning. Judging individuals are highly organized in their thoughts, while perceivers behave more spontaneously.

The MBTI personality categorization scheme defines each of the 4 binary MBTI categories to represent a different aspect of human personality. However, when being combined into 16 personality types, it is known to have a major shortcoming, namely large overlap between the “neighboring” categories, e.g., INTJ and INTP. Given the noisy nature of social media content, we suggest that it might be a good idea to model and predict individual binary MBTI personality traits instead of modeling the overlapping 16-category structure. Therefore, in this work we have adopted a binary personality categorization scheme, leading to four binary classifiers.

### 3.2. PERS multi-source multi-view personality dataset

Our main contribution in this work is a model that predicts user personality from multi-faceted social network data. Thus, we collected the primary dataset for this work from social networks for users whose personality types are somehow known; in this section, we outline data acquisition and feature extraction (preprocessing) for this dataset.

#### 3.2.1. Data acquisition

The data was collected from *Twitter, Facebook*, and *PersonalityCafe* social networks during the time interval from Jan 1, 2018 to Jan 1, 2021. Data acquisition proceeded via the following three steps.

**Figure 1:**
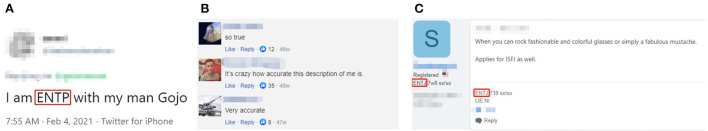
A sample target user from **(A)** *Twitter*, **(B)** *Facebook*, and **(C)** *PersonalityCafe*.

1. **Ground truth collection**. To obtain personality ground truth from *Twitter*, we have downloaded all tweets that contain self-reported personality-related keywords/phrases such as “I'm an **ENTP**” or “I am an **ENTP**” and extracted the personality trait from those phrases. This trait represents the ground truth for each user (see Figure 1A for an example). To harvest *Facebook* ground truth, we have monitored *Facebook* comments under personality test results released on the *16personalities portal* (see Figure 1B for an example). Likewise, to obtain personality-related ground truth from the *PersonalityCafe* forum, we downloaded the users' publicly available self-reported personality traits from their profile pages (see Figure 1C for an example).
2. **User-generated content (UGC) collection**. To establish UGC collection from *Twitter* and *Facebook*, we have downloaded user timelines through *Twitter* REST API^3^ and *Facebook* GRAPH API^4^, respectively. To collect UGC from the *PersonalityCafe* forum, we downloaded posts from the MBTI forum thread.
3. **Data preprocessing**. Since social network data might exhibit significant noise levels and often contains grammatical errors, it becomes necessary to perform data preprocessing prior to the data modeling stage. At the same time, we need to remove direct personality mentions from textual content so that the model will not be able to use personality abbreviations from the post content at the inference stage. To mitigate the above two problems, we have pre-processed our dataset as follows:
  (a) *Data filtering*: to ensure sufficient amount of data available per user for training and inference, we have filtered out users with less than 10 tweets available;
  (b) *Inline label replacement*: for all personality traits, the personality type name was replaced with the TYPE placeholder (e.g., “ENTJ” would be replaced with “TYPE”);
  (c) *Social indicator replacement*: similar to Nguyen et al. (2020), we have further converted emojis into the corresponding descriptive textual strings, removed all non-ASCII words, and normalized the text by replacing user mentions, URLs, hashtags, date-time by the corresponding placeholders as follows: @USER, HTTPURL, HASHTAG, DATETIME.

**Figure 2:**
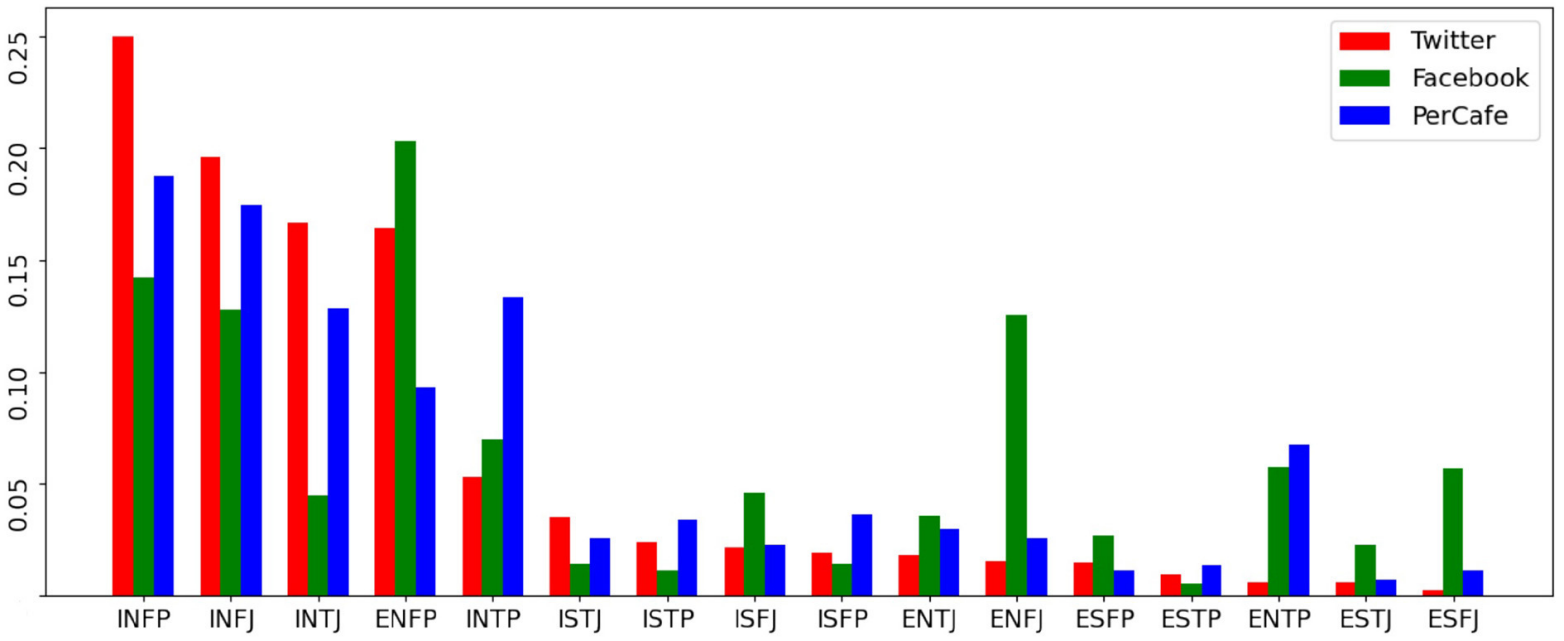
Proportion of personality traits in three data sources.

Tables 1, 2 show the basic statistics of our dataset and statistics across 16 personality labels, and Figure 2 visually reflects the personality label distributions. Figure 2 and Table 2 show that INFP, INFJ, INTJ, ENTP, and INTP are the Top-5 most popular personality labels found in both *Facebook* and *Twitter* datasets, showing the consistency of data distributions across general social networks; this reduces the risk of falling into source-dependent bias during the data modeling stage. At the same time, it is important to note that in the *PersonalityCafe* forum data ENFP, INFP, INFJ, and ENFJ labels dominate the rest. This shows that the personality-related data sources might have a distribution shift toward individuals of certain personality types (different from the general distribution) that tend to participate in such specific personality-related discussions. Therefore, evaluation based on the *PersonalityCafe* dataset must be accomplished independently.

**Table 1:** Dataset statistics.

	**Twitter**	**Facebook**	**PerCafe**
#User	21,305	11,730	3,800
#Posts	8,114,568	2,838,141	621,482
#Images	1,865,562	597,164	-
#Extroversion	5,013	6,243	981
#Introversion	16,292	5,487	2819
#Sensing	2,799	2,300	610
#Intuition	18,506	9,430	3,190
#Thinking	6,743	3,040	1,666
#Feeling	14,562	8,690	2,134
#Judging	9,800	5,528	1,613
#Perceiving	11,505	6,202	2,187

**Table 2:** Distribution of personality traits.

	**PerCafe**	**Twitter**	**Facebook**
INFP	713	5,334	1,665
INFJ	664	4,177	1,498
INTP	508	1,121	814
INTJ	487	3,544	521
ENFP	353	3,496	2,381
ENTP	256	122	671
ISFP	137	413	161
ISTP	127	508	131
ENTJ	113	389	412
ISTJ	98	739	162
ENFJ	96	323	1,468
ISFJ	85	456	535
ESTP	50	200	63
ESFJ	43	52	666
ESFP	43	311	316
ESTJ	27	120	266

#### 3.2.2. Data representation

To facilitate an effective data modeling process, the data needs to be properly represented in the form of feature vectors. Following the best practices described in user profiling literature (Farseev and Chua, 2017b; Rangel Pardo et al., 2018; Khan et al., 2020) we have chosen the following data representation approaches.

**Figure 3:**
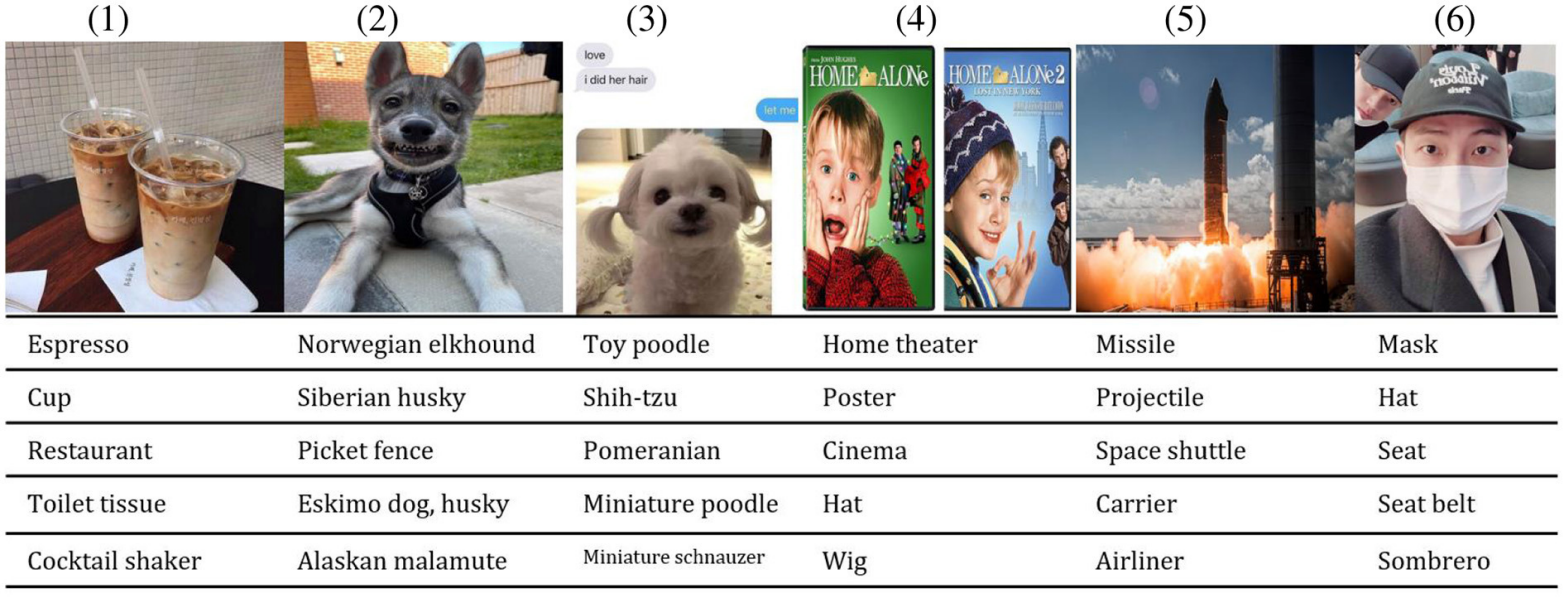
Sample concepts extracted from user images.

1. **Text features**. First, to represent textual data at the user level, for each user all posts were concatenated into the corresponding user-specific “documents.” Second, we used the term frequency-inverse document frequency (TF-IDF) weights to construct the document-term matrix. Finally, we applied the Latent Semantic Analysis model (LSA) (Halko et al., 2011); this simple topic model has been previously shown to lead to significant improvements in performance when applied for user profiling (Daneshvar and Inkpen, 2018). The final dimension of the compressed textual feature vector was set to 100; this number of dimensions has been found empirically during a grid search.
2. **Visual features**. To represent visual data (images), we have automatically mapped each photo into a distribution over 1,000 *ImageNet* (Deng et al., 2009) image concepts via a pre-trained *ResNet-101* model (He et al., 2016). The model predicts a distribution over 1,000 classes (image concepts), and we extract image features as probabilities of these 100 image concepts for every image to represent the user's image preferences. Figure 3 shows several sample concepts extracted from the images in user timeline data; note that sometimes these concepts correspond to different objects on the image (“mask,” “hat,” and “seat belt” on image 6), sometimes they consider the same primary object from different sides (“missile”, “projectile,” and “carrier” on image 5), and other times they “cover” different closely related possibilities (such as different dog breeds in images 2 and 3). We then summed up the predicted concept occurrence likelihoods for each user and normalized the resulting vector element-wise by the total number of images available from the user. In such a way, for each user, we have obtained a 1, 000-sized image concept distribution vector. Similarly to the text modality, we have further applied principal component analysis (PCA) (Jolliffe, 2011) to reduce the dimensionality of the visual feature space to 200.

### 3.3. PERS Rec multi-view dataset

To investigate the impact of human personality traits on the performance of content recommendation systems with social media data, we need a large dataset with the interactions of users and social media posts. In this section, we present the details of such a dataset.

#### 3.3.1. Data acquisition

Inspired by Gelli et al. (2018), we choose *Twitter* as the main source for this dataset. We selected 48 brands with 100 posts each (the threshold of 100 was chosen by us to have sufficient data), thus obtaining a set of 48 brands and 4,800 posts by these brands. Next, we retrieved the list of users who liked these posts as well as their timeline data with the same methodology as in Section 3.2.1. Table 3 shows the basic statistics of the resulting dataset.

**Table 3:** PERS Rec dataset statistics.

**Item**	**Brands**	**Brands posts**	**Inter**	**Users**	**Posts**	**Images**	**Sparsity**
Quantity	48	4,800	330,545	41,901	6,547,342	1,407,775	99.835%

#### 3.3.2. Data representation

To represent the brand posts data, we follow the same data representation approach as for the personality dataset, as outlined in Section 3.2.2.

1. **Text features**. We extracted the TF-IDF features for each post to form the document-term matrix and then applied latent semantic analysis (LSA) (Dumais, 2004) to reduce the textual feature dimension to 100. For a given number of features *k* (in our case *k* = 100), latent semantic analysis applies the singular value decomposition to the document-term matrix *X* (in our case to the matrix of tf-idf weights), obtaining *X* = *UΣV*^⊤^, restricts *U*, Σ, and *V* to *k* top singular values, obtaining the low-rank approximation $X_{k}=U_{k}\Sigma_{k}V_{k}^{⊤}$, and then represents a document **d** as a vector $U_{k}^{⊤}\text{d}\in {\mathbb{R}}^{k}$.
2. **Visual features**. We mapped each of the posts into the distribution of 1,000 ImageNet image concepts *via* the pre-trained ResNet-101 model and applied principal component analysis (PCA) on the image concepts matrix to reduce the visual feature dimension to 200 (Jolliffe and Cadima, 2016). Principal components analysis finds the orthonormal basis of vectors (principal components) **w** that sequentially maximize the variance of data points projected on these components: for a data matrix *X* (in our case, the *N* × 1, 000 matrix of concept distributions), ${\text{w}}_{1}=\arg\underset{||\text{w}||=1}{\max}||X\text{w}|{|}^{2}=\arg\underset{||\text{w}||=1}{\max}{\text{w}}^{⊤}X^{⊤}X\text{w}$, **w**_2_ is found similarly after projecting *X* to the subspace orthogonal to **w**_1_, and so on; to obtain the features, *X* is projected to the first *k* (in our case, *k* = 200) principal components: $\hat{X}=XW_{k}$, where *W*_*k*_ = (**w**_1_, …, **w**_*k*_).

In addition to the method of user data representation described in Section 3.2.2, we have inferred the user's personality representation with the PERS model; our main goal on this dataset is to investigate whether inferred personality traits can bring significant benefits to recommender systems.

## 4. Methods

### 4.1. Social media data

For a user *i*, we are given their multi-view data that consists of the text features, image features, and personality traits. Thus, the dataset can be thought of as the set


(1) $$\begin{array}{r} X=\left\{\left({\text{x}}_{i}^{\text{text}},{\text{x}}_{i}^{\text{image}},{\text{y}}_{i}\right),i=1,2,\ldots ,n\right\}, \end{array}$$

where *n* is the number of users, ${\text{x}}_{i}^{\text{text}}\in {\mathbb{R}}^{100}$ is the vector of text features after LSA dimensionality reduction (see Section 3.3.2), ${\text{x}}_{i}^{\text{image}}\in {\mathbb{R}}^{200}$ is the vector of image features after PCA dimensionality reduction (see Section 3.3.2), and ${\text{y}}_{i}\in {\left\{0,1\right\}}^{4}$ is the vector of four binary personality trait ground truth labels for the *i*th user, one label for each of the four opposing features: **E** vs. **I**, **S** vs. **N**, **T** vs. **F**, and **J** vs. **P**. We formalize personality profiling as a set of four binary classification tasks, so below we describe the framework for one binary classification with label *y*_*i*_∈{0, 1}, and the same process is repeated four times to obtain the final prediction vector ${\hat{\text{y}}}_{i}\in {\mathbb{R}}^{4}$ for every user *i*.

### 4.2. PERS framework

**Figure 4:**
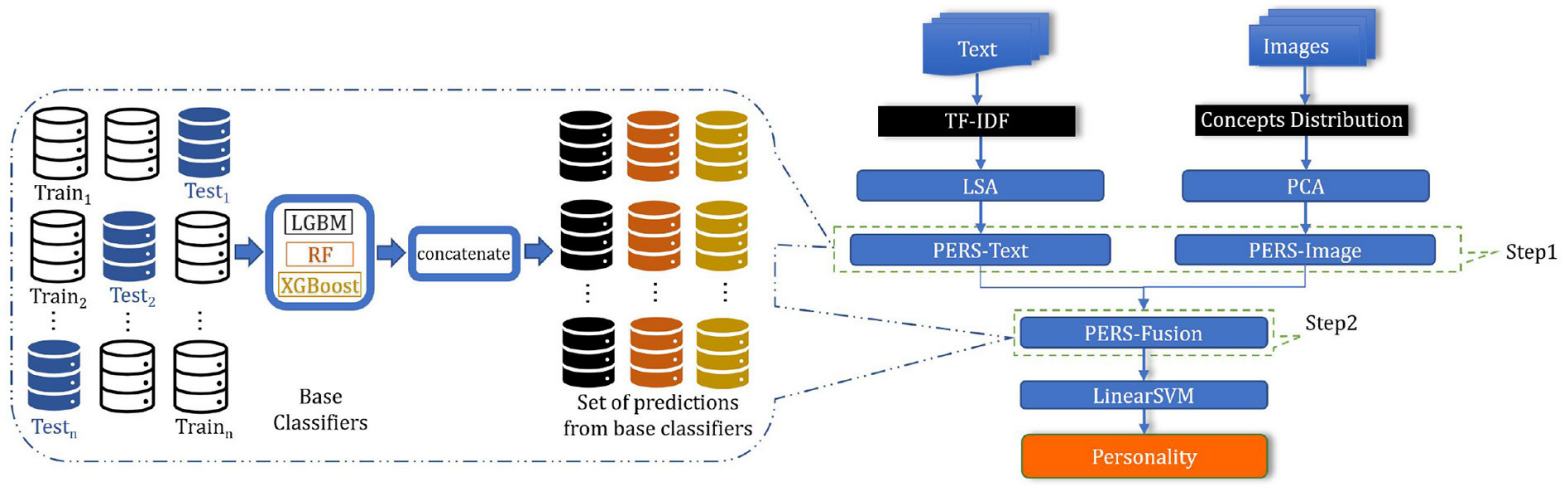
Overview of the PERS framework.

We now can define the PERS framework as a two-step stacked generalized ensemble approach. The main idea of our approach to ensembling is to use *K*-fold cross-validation on the original dataset and use predictions of the *J* classifiers on the *K* test sets as features for training the fusion part. To obtain a vector of personality feature scores, we need to perform four binary classifications, one for each pair of opposing traits. The architecture of the proposed PERS framework is illustrated in Figure 4.

For a binary classification model C operating on inputs **x**∈ℝ^*m*^, we denote by C_*X*_ the model C trained on a training set *X* = {(**x**_*i*_, *y*_*i*_), *i* = 1, 2, …, *n*}, where ${\text{x}}_{i}\in {\mathbb{R}}^{m}$ and *y*_*i*_∈{0, 1}, and denote by ${\text{C}}_{X}\left(X^{\prime }\right)$ the prediction results of C_*X*_ on a test set $X^{\prime }=\left\{{\text{x}}_{i},i=1,2,\ldots ,n^{\prime }\right\}$. We assume that C outputs a probability score for the binary classification label, so ${\text{C}}_{X}\left(X^{\prime }\right)\in {\mathbb{R}}^{n^{\prime }}$ and ${\text{C}}_{X}{\left(X^{\prime }\right)}_{i}\in \left[0,1\right]$.

**Step 1**. In essence, on Step 1 we choose a list of “base” classifiers of size *J*, $C=\left\{{\text{C}}^{\left(1\right)},\ldots ,{\text{C}}^{\left(J\right)}\right\}$, train each *j*th classifier on the training set *X* and use the predictions of these classifiers as features for Step 2. To avoid using the same samples in the training and test set, we do it *via* *K*-fold cross-validation as follows (and as illustrated in Figure 4 on the left):

- split the training set *X* = {(**x**_*i*_, *y*_*i*_), *i* = 1, 2, …, *n*}, where *y*_*i*_ is the personality label and **x**_*i*_ are feature vectors for user *i*, into *K* disjoint subsets *X*_*k*_, denoting also *X*_−*k*_ = *X*\*X*_*k*_;
- train each classifier C^(*j*)^, *j* = 1, …, *J*, on each subset *X*_−*k*_, obtaining ${\text{C}}_{k}^{\left(j\right)}={\text{C}}_{X_{-k}}^{\left(j\right)}$;
- apply each classifier ${\text{C}}_{k}^{\left(j\right)}$ to the corresponding test set *X*_*k*_, obtaining ${\text{C}}_{k}^{\left(j\right)}\left(X_{k}\right)$.

As a result, for each sample **x**_*i*_∈*X* we obtain the predictions of *J* base classifiers (each **x**_*i*_ participates in one test set *X*_*k*_), and these predictions are concatenated to obtain the feature vector ${\text{z}}_{i}\in {\mathbb{R}}^{J}$. We perform the above process separately for text features **x**^text^ and image features ${\text{x}}_{i}^{\text{image}}$, so the final feature vector at the output of Step 1 for user *i* has size 2*J*. This concatenation is denotes as PERS-Fusion in Figure 4.

During inference, on Step 1 we apply all *J* classifiers trained above to the features of a new user *i* and average their predictions.

**Step 2**. To get the final model prediction, we train a meta-classifier *f* on the set of features *Z* = {**z**_1_, …, **z**_*n*_}, ${\text{z}}_{i}\in {\mathbb{R}}^{2J}$, extracted on Step 1, obtaining the final prediction as


(2) $$\begin{array}{r} ŷ=f\left(\text{z}\right) \end{array}$$

for the feature vector **z**∈ℝ^2*J*^ produced as above. Specifically, in this study we have chosen a support vector machine with linear kernel (LinearSVM) as our meta-classifier (Lee et al., 2019).

### 4.3. Base classifiers

To maximize the performance of the PERS framework, it is of crucial importance to choose a set of suitable machine learning algorithms as base classifiers. Previous studies (Farseev et al., 2015; Amirhosseini and Kazemian, 2020; Qi et al., 2020) suggest XGBoost (Chen and Guestrin, 2016), LightGBM (Ke et al., 2017), and random forests (Ho, 1998; Breiman, 2001) to be the top choice base models for user profiling. Their performance on social media data has been reported to beat other baselines, often including state of the art neural models. Therefore, we choose these three approaches as our base classifiers.

#### 4.3.1. Random forest

Random forest is an ensemble learning algorithm that integrates multiple decision trees to make predictions (Ho, 1998; Breiman, 2001). For the classification problem, the prediction result is the vote of all decision tree prediction results. During training, bootstrap sampling is used to construct the training set for each decision tree. When training each node of each decision tree, the features used are also part of the features extracted from the entire feature vector. By integrating multiple decision trees and training each decision tree with different data subsamples and feature components every time, the variance of the model can be effectively reduced.

#### 4.3.2. XGBoost

XGBoost is an effective and scalable gradient boosting machine that has been widely adopted in the machine learning industry over the last decade (Chen and Guestrin, 2016). It is an ensemble model containing a set of classification and regression trees (CART). Given a feature vector **x**_*i*_ and target *y*_*i*_, the XGBoost model can be defined as


(3) $$\begin{array}{r} {ŷ}_{i}=\overset{M}{\underset{m=1}{\sum }}f_{m}\left({\text{x}}_{i}\right),\;f_{m}\in F, \end{array}$$

where *M* is the total number of trees, *f*_*m*_ is the function implemented by the *k*th tree, and *F* is the function space of all possible CARTs.

#### 4.3.3. LightGBM

LightGBM is an improved version of gradient boosting machines that mitigates the “optimal division point search” problem that leads to increased computational complexity on larger datasets (Ke et al., 2017). The problem is solved *via* the following two tricks, reducing the training data size and data dimensionality:

1. *Gradient-based one-side sampling* (GOSS): exclude most of the samples with small gradients and only use the remaining samples to calculate the information gain;
2. *Exclusive feature bundling* (EFB): bundle mutually exclusive features together since they rarely take nonzero values at the same time.

### 4.4. Content recommendation model

To understand the impact of human personality (inferred by the PERS framework) on the performance of content recommendation, it is vital to choose a suitable model to take the advantage of multimodal data that would be able to make good use of new features. We have adopted the Personalized Content Discovery (PCD) model (Gelli et al., 2018) as it learns fine-grained user representation *via* explicit modeling of the user's personality traits and is able to leverage the multi-view data.

PCD is inspired by matrix factorization; it uses a deep neural network to extract an item representation from item features, a different deep neural network to extract a user representation from user features, and finally the triplet ranking loss to train on positive and negative (user, item) pairs. In our case, users are represented as concatenations of text features, image features, and inferred personality traits. The latter are represented as vectors of length 4 with each dimension showing the corresponding MBTI dimension (EI, SN, TF, and JP) as a number from the [0, 1] range (probability inferred by the corresponding binary classifier). Note that we do not change the PCD model itself to account for personality traits in some special way, we are using PCD “as-is” and simply adding personality traits as new features; in the same way, personality traits can be added to other content recommendation models as well, in particular, the ones surveyed in Section 2.

## 5. Evaluation

### 5.1. Baselines

To answer our research questions, we have evaluated the performance of the PERS framework being trained across different data sources and data modality combinations. For all experiments, the dataset was uniformly split into a training set and test set with the ratio of 85:15, with the split preserving the original personality label distributions. To understand the impact of different modalities, data sources, and fusion strategies on the final performance of the model, we have selected the following community-adopted personality profiling baselines (Farseev et al., 2015; Rangel et al., 2015; Buraya et al., 2017):

- **Independently trained base classifiers** (see descriptions in Section 4.3) with respect to each data modality;
- **Early fusion**: base classifiers trained based on the early-fused data modality representations (concatenated feature vectors);
- **Early fusion (PCA 200)**: base classifiers trained based on the early-fused data modality representations with PCA applied after the vector concatenation (the PCA dimension of 200 has been selected empirically via grid search).

To understand the impact of human personality on content recommendation, we selected the following user representations as baselines:

- **Matrix factorization** (MF), the basic approach to collaborative filtering;
- **Neural collaborative filtering** (NCF), a generic approach to recommender systems that can generalize matrix factorization under its framework by replacing the inner product with a neural architecture that can learn an arbitrary function from data;
- **PCD with one-hot user representation**, the PCD model with a one-hot encoding of user representation;
- **PCD with user timeline representation**, the PCD model with each user represented by textual and visual features as described in Section 3.2.2.

### 5.2. Evaluation metrics

Due to the imbalanced distribution of personality labels in our datasets (see Section 3.2.1 for details on the data distributions), for performance evaluation we have adopted the *F*_1,macro_ metric (Farseev et al., 2015), which is the harmonic mean between precision and recall, and the average is calculated per label across all labels. The *F*_1,macro_ metric is formally defined as


(4) $$\begin{array}{r} F_{1,\text{macro}}=\frac{1}{Q}\overset{Q}{\underset{j=1}{\sum }}\frac{2p_{j}r_{j}}{p_{j}+r_{j}}, \end{array}$$

where *p*_*j*_ and *r*_*j*_ are the precision and recall for the *j*th label out of *Q*.

We have further adopted the *Matthews correlation coefficient* metric (Mcor) (Matthews, 1975), as it incorporates both true and false positives and negatives and is generally regarded as a “balancing” metric that can be used even if the classes are of a very different size. The Mcor metric is formally defined as


(5) $$\begin{array}{r} \text{Mcor}=\frac{TP\cdot TN-FP\cdot FN}{\sqrt{\left(TP+FP\right)\cdot \left(FN+TN\right)\cdot \left(FP+TN\right)\cdot \left(TP+FN\right)}}, \end{array}$$

where TP is the number of true positives, TN, the number of true negatives, FP, false positives, and FN, false negatives.

We prioritize the *F*_1,macro_ score as our main evaluation metric for user personality profiling, while the Mcor score plays an auxiliary role for making decisions regarding performance when the *F*_1,macro_ values are marginal.

In order to evaluate the impact of human personality traits on content recommendation, we have chosen the area under curve (AUC), normalized discounted cumulative gain (nDCG), and F1 score as the metrics.

### 5.3. Evaluation across MBTI categories

To evaluate the performance of the PERS framework in a real-world scenario, we have evaluated the limits of PERS performance across *Twitter, Facebook*, and *PersonalityCafe* datasets. Evaluation results are presented in Table 4.

**Table 4:** Evaluation of the PERS framework trained on independent modalities and modality combinations on *Twitter* and *Facebook* datasets.

**Model**	**Twitter**				**Facebook**			
	**EI**	**SN**	**TF**	**JP**	**EI**	**SN**	**TF**	**JP**
**Text** (*F*_1, macro_/Mcor)								
XGBoost	0.80/0.62	0.48/0.08	0.59/0.23	0.61/0.22	0.62/0.25	0.59/0.22	0.55/0.16	0.58/0.17
RF	0.78/0.56	0.56/0.14	0.61/0.22	0.62/0.25	0.61/0.21	0.62/0.26	0.58/0.17	0.58/0.16
LGBM	0.80/0.62	0.56/0.14	0.61/0.24	0.62/0.25	0.62/0.24	0.62/0.24	0.57/0.13	0.58/0.16
**Image** (*F*_1,macro_/Mcor)								
XGBoost	0.46/0.01	0.47/0.03	0.51/0.01	0.54/0.09	0.59/0.19	0.47/0.05	0.49/0.07	0.56/0.14
RF	0.54/0.09	0.52/0.06	0.57/0.14	0.56/0.12	0.59/0.18	0.54/0.08	0.57/0.15	0.57/0.14
LGBM	0.52/0.05	0.52/0.04	0.56/0.11	0.57/0.13	0.59/0.18	0.55/0.11	0.57/0.14	0.56/0.12
**Early Fusion (*F*_**1,macro**_/Mcor)**								
XGBoost	0.8/0.62	0.48/0.06	0.59/0.22	0.59/0.19	0.60/0.21	0.56/0.22	0.54/0.16	0.58/0.17
RF	0.78/0.56	0.54/0.1	0.62/0.24	0.62/0.24	0.64/0.27	0.62/0.24	0.60/0.20	0.57/0.15
LGBM	0.80/0.61	0.55/0.11	0.62/0.25	0.62/0.24	0.62/0.24	0.61/0.22	0.60/0.21	0.60/0.20
**Early Fusion (PCA 200)(*F*_**1,macro**_/Mcor)**								
XGBoost	0.79/0.61	0.48/0.07	0.59/0.23	0.60/0.20	0.57/0.14	0.47/0.04	0.50/0.07	0.55/0.10
RF	0.78/0.56	**0.57/0.14**	**0.63/0.26**	0.62/0.24	0.60/0.21	0.54/0.08	0.58/0.16	0.56/0.13
LGBM	0.80/0.60	0.56/0.11	0.62/0.25	0.62/0.24	0.59/0.18	0.52/0.04	0.56/0.12	0.56/0.13
**PERS Trained with Single Modality (*F*_**1,macro**_/Mcor)**								
Text	0.81/0.62	0.55/0.14	0.62/0.26	0.63/0.28	0.62/0.23	0.62/0.28	0.59/0.21	0.58/0.16
Image	0.53/0.11	0.47/0.05	0.57/0.16	0.59/0.17	0.59/0.17	0.50/0.10	0.56/0.17	0.56/0.13
**PERS Trained with Dual Modalities (*F*_**1,macro**_/Mcor)**								
T+I	**0.82/0.61**	0.54/0.12	**0.63/0.26**	**0.64/0.28**	**0.64/0.28**	**0.63/0.30**	**0.61/0.23**	**0.62/0.21**

The best performance is highlighted in bold.

From the table, it can be seen that after training on the multi-view data from *Twitter*, the PERS framework is able to achieve an industry-level performance of 0.82 *F*_1,macro_ score when predicting the Extroversion-Introversion (EI) personality trait. While the performance obtained for the other three personality categories is significantly lower, ranging from 0.64 *F*_1,macro_ score for Judging-Perceiving (JP) to 0.54 *F*_1,macro_ score for Sensing-Intuition (SN), we still believe that such a promising performance for the EI label indicates the tremendous potential of multi-view social media data used for psychographic discovery and personality profiling. These especially good results for the EI label can be explained by the natural difference of these two human personality categories when it comes to user communication on social platforms: extroverts are known to be much more open to others, while introverts are being more selective and are making decisions at a more conservative pace. Such inspiring results allow us to give a **positive answer to our RQ1**; these results open up a wide range of new research directions related to personality profiling and Multi-View learning.

As for the other two datasets, an interesting finding comes from the results presented in Table 5, where PERS demonstrates breakthrough performance based on the *PersonalityCafe* dataset; it shows the best overall *F*_1,macro_ scores when predicting all four binary MBTI categories. This result can be explained by the nature of the *PersonalityCafe* dataset, where users reveal their behavioral differences on purpose and are therefore often biased toward particular social behavior concepts. Such results also **confirm our positive answer to RQ1** and allow us to conclude that indeed the nature of a data source and social network use patterns are of crucial importance when solving the multi-view cross-media personality profiling problem.

**Table 5:** Evaluation of the PERS framework trained on the text modality on *PersonalityCafe*.

**Text**(***F***_**1, macro**_/Mcor)				
**Model**	**EI**	**SN**	**TF**	**JP**
LGBM	0.69/0.39	0.74/0.50	0.80/0.61	0.73/0.47
XGBoost	0.69/0.41	0.69/0.42	0.79/0.60	0.73/0.46
RF	0.65/0.29	0.67/0.33	0.74/0.53	0.69/0.36
**PERS**	**0.71/0.43**	**0.74/0.51**	**0.81/0.61**	**0.74/0.49**

### 5.4. Evaluation across different modalities

First, we have investigated the contribution of different data modalities toward personality profiling performance and its integration ability. An interesting observation comes from the cross-modal experimental results presented in Table 4: the PERS framework has performed 2% better than other single-source baselines for all but SN binary labels being trained on *Twitter* and *Facebook* datasets. Another interesting observation can be made from the modality combination results, where by training with both text and image data PERS is able to outperform by more than 1% not just other unimodal classifiers but also all early-fused baselines.

The above findings suggest that the introduction of multimodality into user profiling could serve as a powerful booster of model performance. Such observation could be explained by the richness of visual data when reflecting user preferences, which serves as a beneficial supplement for the basic textual data modality at the data modeling stage. The latter finding **positively answers our RQ2** by emphasizing the important role of multimodal data learning for personality profiling applications.

Finally, let us also highlight an interesting observation that comes from single-modal evaluation results (see Table 4). It is important to note that, in case when we are learning from a unimodal source, PERS trained on the text modality performs best across all personality labels, with improvements ranging from 0.02 to 0.28 in terms of the *F*_1,macro_ score. This effect can be easily explained by the quantitative domination of text data over the visual modality (recall Table 1). Another potential reason for this result could be the high level of noise in user-generated visual data, where the images are less strict in terms of perspective and object positioning as compared to professional photos. Moreover, such visual content often includes objects that might not directly reflect the semantics of the data and therefore might be not accurate in representing the author's personality. To this end, such hypothesis also aligns well with our chosen visual data representation approach, where the *ImageNet* concept distribution might be simply too general for personality profiling tasks, as opposed to, for example, demographic profiling (Farseev et al., 2015). As a result, we are positive in our general recommendation to use personality traits to supplement content recommendation systems even if the traits themselves are not available and have to be inferred, as is usually the case in practice.

### 5.5. Evaluation across different sources

Next, let us examine the impact of the social media data origin on personality user profiling performance, so that an industry guideline can be established for future research.

As the text modality has participated in all three data sources, we begin with PERS performance on text data. Tables 4, 5 indicate that the PERS framework being trained on *Twitter* dataset outperforms the PERS framework on *Facebook* and *PersonalityCafe* datasets by more than 0.19 *F*_1,macro_ score in predicting the EI label. On the other hand, when it comes to the SN label, *Twitter*-trained PERS was not able to outperform *Facebook* and *PersonalityCafe* data, staying behind by 0.2 and 0.11 *F*_1,macro_ score, respectively. Finally, the PERS performance on TF and JP labels based on *Twitter* text data was found to be better than with *Facebook* data by 0.03 and 0.05 *F*_1,macro_ score, respectively, but considerably worse than the *PersonalityCafe* data: by 0.19 and 0.11 *F*_1,macro_ score, respectively.

The superiority of *Twitter* in predicting the EI label could be explained by the differences of the “energy source” for extroverted and introverted personality types. Specifically, according to Martin (1997), extroverts prefer to source their life energy from active involvement in events and engaging in different activities, while introverts often prefer doing things alone, obtaining their energy from dealing with ideas, pictures, memories, and reactions that are inside their mind. Similarly, in the digital world it can be seen that on *Twitter* both personality types are able to express themselves fulfilling both their enjoyment (ENJ) and observation/learning (LEN) needs, while for *Facebook* the ENJ factor got fulfilled proportionally for a smaller number of individuals, affecting the overall user base distribution (Syn and Oh, 2015). Correspondingly, *Twitter* and *PersonalityCafe* datasets are diverse enough to differentiate EI personalities and allow for higher prediction scores as compared to *Facebook*-based predictions. This observation is also supported by our data distribution (see Section 3.2.1), where *Twitter* and *PersonalityCafe* datasets are clearly skewed toward introverts, providing more data for PERS to learn on how this personality type direct their energy and make decisions. The latter aspect is important as it is known that extroverts might generate substantially more UGC as compared to introverts (Syn and Oh, 2015), and therefore it is crucial to have sufficient content generated by introverts for mutually consistent and comprehensive learning from the data.

At the same time, the opposite picture can be noticed for the SN label results. Again, there is a “low-hanging” explanation of this phenomenon: for both *Twitter* and *Facebook*, the SN personality is distributed with a clear shift toward the intuitive personality type, and both datasets are short on sensing individuals. Despite reflecting the real life distribution, this data property also entails a possible technical issue: the data variation may be insufficient, limiting the model when it comes to learning the sensing and intuitive user personas. Considering that *Facebook* and *PersonalityCafe* datasets have more sensing personality types identified, it is reasonable to assume that this is also the reason why PERS has performed better on these latter two sources as compared to *Twitter*. To the end, a more “sensing” *Facebook* can also be explained by the fact that *Facebook* is mainly treated nowadays as a communication tool, so people land there for fulfilling their daily communication needs, while *Twitter* more often serves as a source of inspiration, attracting more intuitive individuals (Syn and Oh, 2015).

Next, let us compare the visual modality performance. Here, the first thing that jumps to attention is that the image data modality has performed similarly for the cases of TF and JP labels for both *Twitter* and *Facebook* sources, but at the same time *Facebook* performed better for EI and SN labels with 0.06 and 0.03 *F*_1, macro_ score performance improvement, respectively. As we have mentioned earlier, both personality categories are very different in the way they direct the energy and perceive the external world (Martin, 1997), and therefore the data diversity introduced by incorporating the visual modality is of crucial importance for PERS performance. Since *Twitter* is a “less visual” data source as compared to *Facebook*, and also its data distributions are less balanced (as discussed above) for both personality labels, it is reasonable to assume that these two factors might explain the superiority of *Facebook* data over *Twitter* in the visual modality in our particular case.

Finally, note that PERS trained only based on textual data from the *PersonalityCafe* forum outperforms the results obtained from both *Twitter* and *Facebook* data by at least 0.1 *F*_1, macro_ score. This finding can be explained by the precise focus of *PersonalityCafe* on the topic of personality, which provides additional meaningful data descriptors that can be utilized by PERS to improve its personality inference score.

Backed up by all observations above, we can now **give an answer to RQ4** by highlighting the drastic difference of social media data sources when used in automated personality profiling, which is caused by the way different personalities engage into social network activities. As a practical recommendation, we suggest that online marketing practitioners take these differences into account when designing advertisements: different personality traits suggest different ways of reaching the person with ad, and we expect significant improvements on the initial stages of the marketing funnel if the ads are tailored to the users' personality traits.

### 5.6. Evaluation on the impact of human personality on content recommendation

Results of content recommendation experiments are given in Tables 6, 7. First, in order to understand the complexity of our content recommendation task, we evaluated the performance of baseline models on the PERS Rec dataset (see Section 3.3). The first interesting finding can be seen in Table 6, where the PCD model achieved the best performance across all metrics; on the contrary, the performance of the matrix factorization (MF) model is far from that of the PCD. This finding indicates that in order to serve personalized content with a recommender system, to improve the performance it is crucial to learn a fine-grained user representation. According to the performance of the MF model, we can also say that it is a hard problem to recommend content from only user-item interactions, which can be explained by the sparsity of the interaction distribution and content diversity.

**Table 6:** Performance evaluation of content recommendation models.

**Model**	**AUC**	**nDCG@10**	**nDCG@50**	**F1@10**	**F1@50**
MF	0.8318	0.0115	0.0272	0	0.006
NeuCF	0.847	0.079	0.119	0.0457	0.0817
**PCD**	**0.88**	**0.08**	**0.123**	**0.048**	**0.09**

**Table 7:** Results of the feature ablation experiment.

**Feature combination**	**AUC**	**nDCG@10**	**nDCG@50**	**F1@10**	**F1@50**
One-hot	0.76	0.042	0.07	0.013	0.0232
Post	0.889	0.077	0.113	0.045	0.089
**Post+pers**	**0.897**	**0.087**	**0.13**	**0.051**	**0.095**

But this only goes to show that PCD is better than MF on our dataset; what about personality features? We have studied this question with experiments summarized in Table 7: the performance of PCD further improves across all metrics when we supplement it with user personality features; recall that these are not ground truth personality traits but features predicted by the PERS framework, which corresponds to the realistic use case of our model. **This finding answers RQ3 positively** and goes in line with previous research that shows how users with the same personality traits share similar content preferences (Debra and Worthington, 2003; Dunn et al., 2012).

## 6. Limitations and future work

Although PERS outperforms baselines for all binary personality inference tasks, after combining all binary predictions together the resulting label might often mismatch the actual user's MBTI personality score. Therefore, we recommend that only binary personality predictions (such as EI prediction for the *Twitter* dataset) are leveraged in a real world setting with the existing PERS framework.

This leads us to an obvious line for further work: it is evident that new data source-specific multi-view learning approaches need to be developed (Farseev and Chua, 2017b; Farseev et al., 2017); in such approaches, personality profiling can leverage additional multi-view data representations such as avatars (Gao et al., 2013), sensor data (Farseev and Chua, 2017a), and others, thus mitigating specific issues arising from the difference of communication styles across different social avenues. The development of such models and their application for content generation or recommendation services will be the focus of our future research.

Finally, the influence of content recommendation algorithms on social media platforms should not be ignored since it may bias the user's perceived content and reduce real world performance. Hence, in order to better understand the user's content preferences it would be interesting to develop novel approaches to recommendations that minimize this impact.

## 7. Conclusions

In this work, we have developed and presented a novel framework for automated human personality profiling that draws across multiple data modalities and social networking sites such as *Facebook, Twitter*, and *PersonalityCafe*. Our proposed personality profiling framework, called PERS, demonstrates state of the art performance and outperforms both single-source and multi-source baselines. With our cross-social evaluation, we have also shown that different social networking platforms exhibit different distinct user communication and usage patterns, which in turn affects user profiling model performance and shows that profiling needs to be treated with care for skewed datasets. Finally, to facilitate future research in this exciting direction we have released our new large-scale cross-social multi-view personality profiling dataset and supplemented it with the corresponding statistics and analytics for the community use.

## Data availability statement

The datasets presented in this study can be found in online repositories. The names of the repository/repositories and accession number(s) can be found below: https://pers.azurewebsites.net/.

## Author contributions

AFa and QY conceived the presented idea, developed the theory, and performed data analysis. AFa and SN verified the proposed methods. SN encouraged QY to investigate the impact of personality traits on the performance of content recommender system. All authors discussed the results and contributed to the final manuscript.

## Funding

This work of QY and AFa shown in Sections Data and feature extraction, Methods, and Evaluation was funded by the Russian Science Foundation, Grant No. 22-11-00135 (https://rscf.ru/en/project/22-11-00135/). The work of SN shown in Sections Introduction and Related work was also supported by the grant of the Ministry of Science and Higher Education of Russia for World Level Research Centers, Grant No. 075-15-2022-289.

## Conflict of interest

Authors QY, AFa, and SN were employed by SoMin.AI. The remaining author declares that the research was conducted in the absence of any commercial or financial relationships that could be construed as a potential conflict of interest.

## Publisher's note

All claims expressed in this article are solely those of the authors and do not necessarily represent those of their affiliated organizations, or those of the publisher, the editors and the reviewers. Any product that may be evaluated in this article, or claim that may be made by its manufacturer, is not guaranteed or endorsed by the publisher.

## Footnotes

^1^ https://www.personalitycafe.com/

^2^PERS Multi-Source Multi-View Personality Dataset, https://pers.azurewebsites.net.

^3^ https://developer.twitter.com/

^4^ https://developers.facebook.com/
